# Novel insights into congenital surfactant dysfunction disorders by in silico analysis of ABCA3 proteins

**DOI:** 10.1007/s12519-022-00645-y

**Published:** 2022-11-20

**Authors:** Guo-Liang Xiao, Yuan Gao, Hu Hao, Tao Wei, Chun Hong, Yue Wang, Ying-Yi Lin, Xiu-Fang Chi, Ying Liu, Hong-Yi Gao, Chuan Nie

**Affiliations:** 1 Department of Neonatology, Guangdong Women and Children Hospital, Guangzhou, 511442 China; 2 Guangdong Neonatal ICU Medical Quality Control Center, Guangzhou, 511442 China; 3 Department of Marine Science, College of Oceanography, South China Agricultural University, Guangzhou, China; 4 Department of Neonatology, The Sixth Affiliated Hospital of Sun Yat-Sen University, Guangzhou, China; 5 Department of Bioengineering, College of Food Science and Institute of Food Biotechnology, South China Agricultural University, Guangzhou, China; 6 Research Center for Micro-Ecological Agent Engineering and Technology of Guangdong Province, Guangzhou, China; 7 Department of Thoracic Surgery, Guangdong Women and Children Hospital, Guangzhou, China; 8 Department of Pathology, Guangdong Women and Children Hospital, Guangzhou, 511442 Guangzhou China

Surfactants produced by type II alveolar epithelial cells (AT2 cells) are usually present in inclusion organelles called lamellar bodies (LBs). The ATP-binding cassette subfamily A member 3 (ABCA3) transporter primarily exists in AT2 cells and is generally considered to be one of the critical regulators of biogenesis of LBs and surfactant metabolism in the lungs [1–4]. *ABCA3* mutations are the most common cause of congenital surfactant dysfunction disorders (CSDDs), resulting in fatal neonatal respiratory distress and pediatric or adult interstitial lung disease [3, 5–7]. More than 200 disease-associated *ABCA3* variants have been identified in symptomatic infants and children [8]. The estimated prevalence of deleterious *ABCA3* mutations in the population is 1/70–1/33, with a predicted disease incidence of 1/20,000–1/4400 [7].

CSDD caused by *ABCA3* mutations lacks specific therapies, and patients are mostly subjected to ineffective conventional treatments [9]. A combination of hydroxychloroquine, corticosteroids, and azithromycin may improve symptoms and delay disease progression in some cases [10–14]; however, evidence-based guidelines are currently lacking. Lung transplantation appears to be a promising way to treat respiratory distress syndrome due to ABCA3 defects [8, 15]. Nevertheless, finding a suitable lung donor in the neonatal period is a considerable challenge for patients. Thus, gene therapy may be a new method for treating these diseases [3]. Therefore, it is crucial to understand the genetic characteristics and pathogenic mechanisms of ABCA3.

The mechanisms underlying compound heterozygosity remain unclear. Since compound heterozygosity mutations play an essential role in the currently reported cases, additional biophysical analyses are needed to predict the effect of gene mutations on the structure and function of the ABCA3 protein, such as homology modeling or 3D model construction for visualization of the target gene sequence.

In general, comprehensive functional analysis, by studying how mutations affect protein variant functions, is essential for understanding the molecular pathogenesis of CSDD and interpreting their clinical significance. This study reports a novel compound heterozygous mutation caused by two missense mutations. In silico analysis elucidated the effects of these novel compound heterozygous variants on protein function using 3D structures. Furthermore, computational analysis of the wild-type and variant proteins revealed the harmful nature of these mutations. The findings of this study can help increase the understanding of compound heterozygous mutations in *ABCA3*. In addition, through impact and domain function analysis before and after amino acid mutation, we can better understand how amino acid substitutions contribute to disease occurrence.

The male neonate was born to a 30-year-old mother (gravida 1, para 1) by vaginal delivery at the 41st week of gestation with 3300 g birth weight. The Apgar scores were 9, 10, and 10 at 1, 5, and 10 minutes, respectively. Two hours after birth, the infant presented with a weak cry, shallow breathing, hypotonia, and cyanosis, and oxygen saturation decreased to 65%–72%. After mechanical ventilation with 60% oxygen and a mean airway pressure of 15 mmHg, oxygen saturation increased to 90%. Cardiac ultrasonography showed an atrial septal defect (4.0 mm), patent ductus arteriosus (4.2 mm), and persistent pulmonary hypertension (75 mmHg). Arterial blood gas showed pH 7.281, pO_2_ 26.2 mmHg, pCO_2_ 67.4 mmHg, and HCO_3_^−^ 25.8 mmol/L. Nitric oxide (NO) inhalation improved persistent pulmonary hypertension, and the oxygen requirement decreased to 40% with SpO_2_ 92%. Consecutive chest radiographs revealed diffuse lung disease similar to neonatal hyaline membrane disease (Supplementary Fig. 1). In addition, chest X-ray examination did not provide valuable information to assist in the diagnosis. Computed tomography revealed blurred bronchovascular bundles and decreased transparency in both lungs. In addition, we detected a large patchy high-density shadow in the anterior segment of the left upper lobe of the right lung and on the lower lobe of both lungs (Supplementary Fig. 2). Pulmonary hypertension improved to 35 mmHg after NO inhalation, according to the cardiac ultrasonography findings on day 3. However, hypoxemia and carbon dioxide retention persisted after ventilation with high-frequency oscillatory ventilation or volume-guaranteed pressure controlled (tide volume 6 mL/kg). High-throughput genetic sequencing technology detected *Ureaplasma* infection in the blood and sputum, which was cleared one week after azithromycin treatment.

However, the infant’s condition did not improve significantly. The peak pressure of respiratory support must consistently be maintained above 22 cmH_2_O to maintain a tidal volume of 6 mL/kg, and carbon dioxide retention was not markedly improved. Therefore, whole-exome sequencing (WES) and left lung biopsy via video-assisted thoracoscopic surgery (VATS) were performed on day 21, with the parents’ informed consent and ethical review, to collect lung tissue for pathological examination. Uneven inflation and slightly scattered white granules appeared on the surface of the left lung during the operation (Supplementary Fig. 3). Evidence-based guidelines for hydroxychloroquine use are currently lacking, and we were unable to obtain parental consent for hydroxychloroquine administration. The infant died of respiratory failure at the age of 40 days, and his parents declined autopsy for him.

The infant underwent VATS according to standard protocols for collecting lung tissues. Specimens from the upper and lower lobes of the left lung were formalin-fixed, paraffin-embedded and investigated by conventional histology and immunohistochemistry.

We collected infant and parent blood samples for WES. Genomic DNA from peripheral blood leukocytes derived from the proband was extracted using the QIAamp DNA blood midi kit (Qiagen, Germany). First, 1 μg of each genomic DNA sample was fragmented by sonication and purified to yield 200–300 bp fragments. Second, paired-end adapter oligonucleotides from Illumina (San Diego, California, USA) were ligated into shared genomic DNA. Five hundred nanograms of these tailed fragments were then hybridized to the probe library of SureSelect human all Exon V6 (Agilent, Germany). Finally, the enrichment libraries were sequenced on an Illumina NovaSeq 6000 sequencer (Illumina) as 150 bp paired-end reads.

After sequencing, reads were aligned to the human reference genome (GRCh37/hg19) with the Burrows-Wheeler aligner [16], and potential duplicate paired-end reads were removed using the genome analysis toolkit (GATK) v.4.2.0.0 (https://github.com/broadinstitute/gatk/releases/tag/4.2.2.0). GATK v.4.2.0.0 was used to obtain base quality score recalibration, indel realignment, single-nucleotide variant, and indel discovery. Genotyping was performed using standard hard filtering parameters [17].

Low-quality variants were flagged and excluded from subsequent analyses. Bamdst v.1.0.9 (https://github.com/shiquan/bamdst) was used to assess the clean data coverage of each sample using default parameters. All variants identified in the affected individuals were annotated with databases, such as refGene (https://www.refgene.com), Avsnp150 (https://www.ncbi.nlm.nih.gov/snp/), gnomAD211 (http://gnomad-sg.org/), ClinVar (https://www.ncbi.nlm.nih.gov/clinvar/), dbnsfp41a (https://sites.google.com/site/jpopgen/dbNSFP), Intervar (https://wintervar.wglab.org/) by snpeff5.0d (https://sourceforge.net/projects/snpeff/), and annovar 2020 Jun (https://annovar.openbioinformatics.org/en/latest/). Candidate mutational events were then inspected using an integrative genomics viewer [18]. The resulting variants were excluded when their frequency exceeded 1/100 in Genome Aggregation (GnomAD). Variants were correlated with patient phenotypes and the results of clinical investigation. All variants were classified according to the American College of Medical Genetics and Genomics guidelines (ACMG) standards and guidelines.

The structural model of wild-type ABCA3 was obtained from the UniProt database (https://www.uniprot.org/niport/Q99758). Homologous modeling using SWISS-model (https://swissmodel.expasy.org/interactive) and AlphaFold2 (https://github.com/deepmind/alphafold2) assisted by Google’s Colab (https://colab.research.google.com) arithmetic platform were applied to obtain a relatively accurate model. PyMol (https://pymol.org/2/) was used to label relevant residues. Because of the internal storage size limitation (RAM = 13 GB), we built only a partial model.

MutPred2 (http://mutpred.mutdb.org/) is a machine learning-based method and software package [19] that integrates genetic and molecular data to probabilistically infer the pathogenicity of amino acid substitutions. It is a sequence-based model that utilizes a methodology based on recent machine learning advances. It is trained based on unlabeled positive data and combines prior and posterior probabilities. This is achieved by providing general pathogenicity predictions and ranked lists of specific molecular alterations that may affect the phenotype. In this study, MutPred2 was used to predict and analyze the structure–function relationship of the ABCA3 protein before and after mutation of the two identified amino acid sites.

Histological examination using hematoxylin–eosin (H&E) staining revealed collapse and enlargement of alveoli, increased fibers in the alveolar basement membrane, serous exudate and tissue reaction in the alveoli cavity, angiodysplasia, and inflammatory cell infiltration (Supplementary Fig. 4a–d). Immunohistochemical examination showed strong cytokeratin (CK) expression in alveolar epithelial cells but weak cluster of differentiation (CD) 31 and CD34 expression in pulmonary vascular endothelial cells, which indicated pulmonary vascular dysplasia and widened alveolar septa. In addition, the expression of CD68 in alveolar monocytes revealed inflammatory cell infiltration. We also observed alveolar fibrin deposition by Masson and periodic acid-Schiff (PAS) staining (Supplementary Fig. 4e–j).

Two novel *ABCA3* mutations were found in the compound heterozygosis of the infant. Heterozygous variations in the *ABCA3* gene, c.1142T > G and c.731G > T, have not been previously reported and are unavailable in the GnomAD (http://gnomad.broadinstitute.org) and Exome Aggregation Consortium (ExAC) (https://exac.broadinstitute.org) databases. The c.1142T > G variant is located in exon 11 of *ABCA3*, resulting in p.Leu381Arg. The c.731G > T variant was located in exon 8 of *ABCA3*, resulting in p.Arg244Met. Next-generation sequencing results revealed compound heterozygous mutations of the *ABCA3* gene, with the c.1142T > G mutation originating from the mother and the c.731G > T mutation inherited from the father (Fig. 1). However, the parents had no signs of respiratory distress, even though they carried a distinct mutation in *ABCA3*. Therefore, according to the ACMG [20], these two mutations were classified as variants of uncertain clinical significance.

**Fig. 1:**
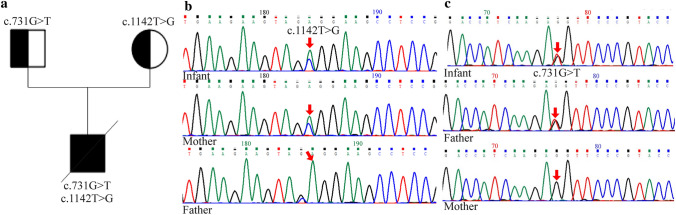
ATP-binding cassette A3 (*ABCA3*) gene mutation analysis. **a** Family pedigree. A novel *ABCA3* compound heterozygosity mutation inherited from the parents. **b** Gene analysis of *ABCA3* in the infant and his parents. Infant: T-to-G substitution in exon 11 (c.1142T > G); infant’s mother: A-to-G heterozygous mutation in exon 11(c.1142T > G); infant’s father: no mutation in exon 11. **c** Gene analysis of *ABCA3* in the infant and his parents. Infant: G-to-T substitution in exon 8 (c.731G > T); infant’s father: G-to-T substitution in exon 8 (c.731G > T); infant’s mother: no mutation in exon 8

Owing to the limitation of the hash rate, the confidence of the calculated model constructed by SWISS modeling is low (global model quality estimate = 54%). Even with the help of Google’s Colab, only 67.3% and 70.58% of residues were involved in the structural modeling of p. Leu381Arg (L381R) and p. Arg244Met (R244M) by AlphaFold2 and SWISS modeling, respectively.

The structure of ABCA3 predicted using the online tool AlphaFold2 indicated that residue 381 was located on the surface of the protein (Fig. 2a, b). The 3D structure in the cartoon format showed that residue 381 was in the middle of an α-helix (Fig. 2c, d). The mutant L381R did not differ substantially at the structural level compared to the wild type. The structure of ABCA3 showed that two caps covered the head and tail of the protein. Residue 244 was not observed on the surface of ABCA3 (Fig. 2a, b). The 3D structure in the cartoon format with a smaller design indicated that it was located at the end of a β-sheet inside the protein (Fig. 2e, f).

**Fig. 2:**
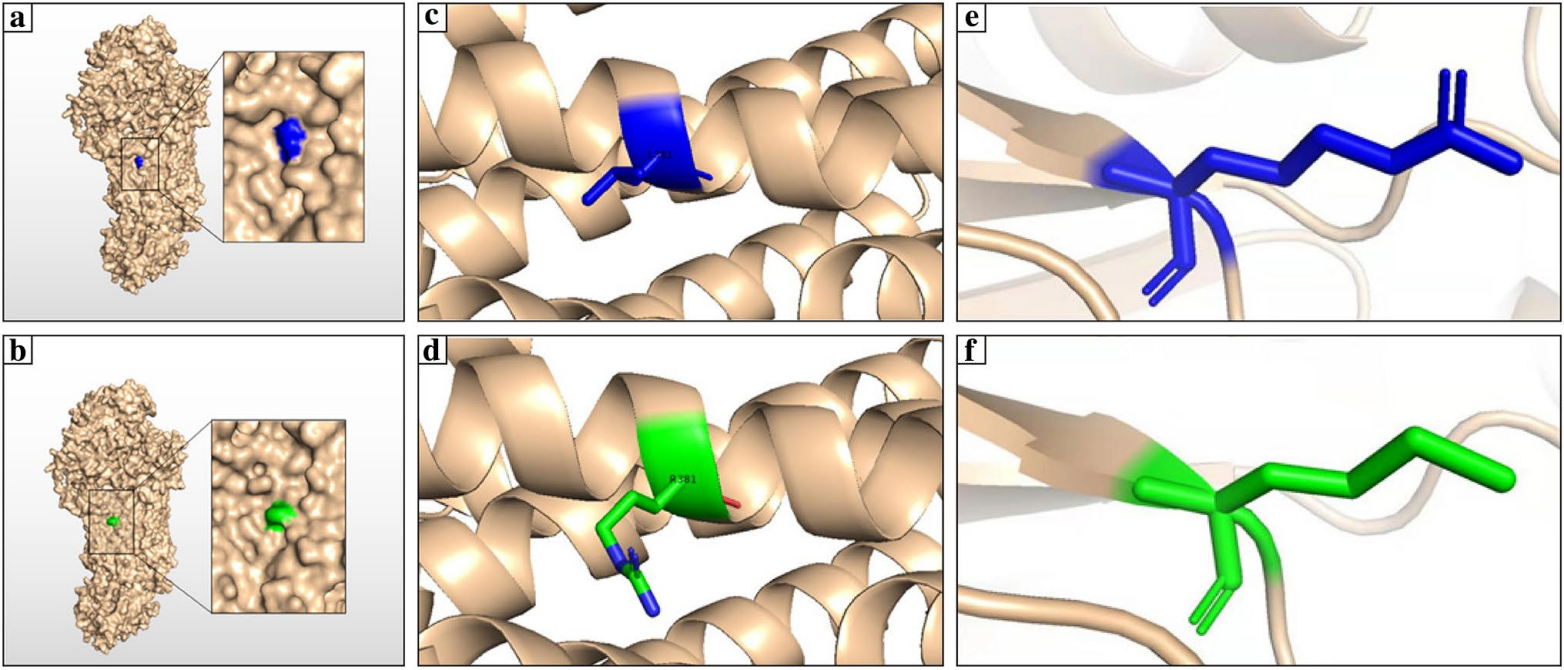
Modelling of AIP-binding cassette A3 (ABCA3) wild type and mutant-type protein. **a**, **b** Three-dimension (3D) structure of the ABCA3 protein. Residue 381 was marked in blue, and the structure of mutant L381R was marked in green. **c**, **d** 3D structure showing before and after mutation of residue 381 (L381R) in cartoon format. Wild type was labeled in blue, and the mutant was labeled in green; the mutant L381R protein did not reveal any structural changes on a smaller scale as well as compared to that for the wild type. **e**, **f** 3D structure showing before and after mutation of residue 244 (R244M) in cartoon format. Wild type was labeled in blue, and the mutant was labeled in green. The 3D structure in the cartoon format with a smaller design indicated that it was located at the end of a β-sheet inside the protein

By reviewing relevant data [21], based on the structural model and delineation of functional regions, we localized and functionally analyzed the structural domains, where the two mutations in this study were located (Fig. 3a). R244M was found in the extracellular domain 1 (ECD1) region, an extracellular structural domain with varying lengths among different members of the ABC family and with associated instability. In contrast, the L381R mutation was primarily located in the transmembrane TM 5 part of transmembrane domain 1 (TMD1), a transmembrane structural domain that is in contact with the cytoplasmic end. At the same time, the TM5 portion of the TMD1 structural domain forms a sizable hydrophobic cavity together with other transmembrane proteins. ABCA3 is primarily distributed on the boundary membrane of the lamellipodia in AT2 cells. To envision its distribution location and transmembrane mode, we used Biorender (https://biorender.com) to draw a 2D distribution schematic of the cell membrane (Fig. 3b).

**Fig. 3:**
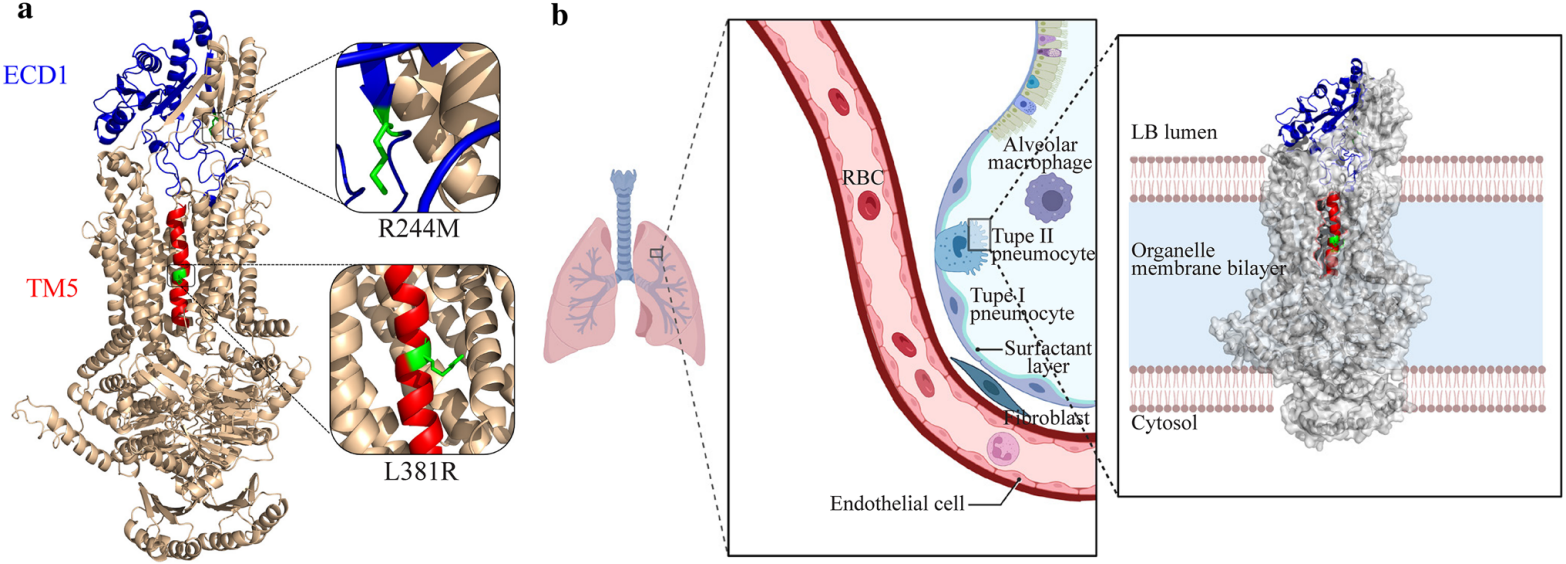
Positions of the structural domains on the ABCA3 protein, where the two mutations are located. **a** Positions of the structural domains on the ABCA3 protein, where the two mutations are located are marked in cartoon format in blue and red for the ECD1 structural domain (blue) and TM5 structural domain (red), respectively. To clearly understand the position of the mutations on the structural domains, we have locally zoomed in on the two mutated amino acids, and the mutated amino acids are indicated in green. **b** Location of ABCA3 in vivo and its possible distribution across the membrane. The structural domains, where the two mutations are located are shown in cartoon format, where ECD1 is shown in blue, TM5 is shown in red, and the other unmutated structural domains are shown in silver surface format. *ABCA3* ATP-binding cassette A3, *ECD1* extracellular domain 1, *TM* transmembrane

We predicted the pathogenicity of the two loci before and after mutation using MutPred2. Relevant analytical results were obtained by introducing the mutation of two loci, R244M and L381R, with the complete amino acid sequence of ABCA3 in the calculation of MutPred2. The results were interpreted in conjunction with the previous delineation of the function and position of the structural domains.

The general pathogenicity score of R244M was 0.751, which is above the pathogenicity threshold of 0.5. This result indicated that the R244M substitution has great potential to produce relevant pathogenic effects on the organism (Table 1). The possible effects were divided into three main areas (in the order of likelihood of occurrence calculated by MutPred2): (1) alteration of the originally ordered interface (*P* value = 8.8 × 10^−3^, possibility 33%); (2) gain of loop (*P* value = 4.9 × 10^−3^, probability 30%); (3) alteration of transmembrane protein structure and function (*P* value = 1.0 × 10^−3^, probability 26%).

**Table 1:** Prediction results of R244M in MutPred2

Molecular mechanisms	Probability	*P* value
Altered ordered interface	0.33	0.008
Gain of loop	0.30	0.005
Altered transmembrane protein	0.26	0.001

Mutations in the L381R locus were also identified in this study. The general pathogenicity score of L381R was 0.808, which was higher than the pathogenicity threshold of 0.5. In terms of molecular mechanism changes, the L381R mutation also has numerous implications (Table 2) divided into two main aspects (in order of likelihood of occurrence). The first was the alteration of transmembrane protein structure and function (*P* value = 4.5 × 10^−3^, 21% probability). The overall structure of the ABCA3 protein L381R before and after the mutation was generated using a self-optimized prediction method (SOPMA) with alignment, online software for computational analysis of protein secondary structure (Table 3). Before the mutation, the percentages of α-helix, β-turn, and random coil were 46.01%, 5.69%, and 32.16%, respectively. After the mutation, the percentage of α-helices decreased by 0.12%, β-turns decreased by 0.06%, and random coils increased by 0.18%, 45.89%, 5.63%, and 32.34%, respectively. The second effect in order of occurrence likelihood is the sulfation deletion of complex 386 (Tyr) (*P* value = 0.05, probability 1%).

**Table 2:** Prediction results of L381R in MutPred2

Molecular mechanisms	Probability	*P* value
Altered transmembrane protein	0.21	0.005
Loss of sulfation at Y386	0.01	0.050

**Table 3:** Statistics of the percentage of secondary structure of proteins that have changed of L381R in self-optimized prediction method (SOPMA)

Secondary structure name	Before mutation (%)	After mutation (%)	Amount of change (%)
Alpha helix	46.01	45.89	− 0.12
Beta turn	5.69	5.63	− 0.06
Random coil	32.16	32.34	0.18

*ABCA3* mutations are the most common cause of genetic surfactant dysfunction disorders, resulting in loss of function of phospholipid transporters involved in pulmonary surfactant function, leading to fatal neonatal respiratory distress [8, 15, 22]. *ABCA3* mutations include multiple forms, including missense mutations, splice sites, insertions, and deletions [23]. These mutations are autosomal recessive and result in distinct clinical phenotypes [8]. Although studies have discovered more than 200 diseases associated with *ABCA3* variants in symptomatic infants and children, approximately ¾ of the reported cases of pathogenic *ABCA3* mutations are associated with compound heterozygous mutations [3]. An overview of some previously reported *ABCA3* mutations is presented in Table 4. Although the mechanisms underlying this phenomenon have not been well-defined [3, 9, 23, 24, 25], whether these mutations are dominant-negative remains to be elucidated. It has been suggested that exposure to certain harmful environmental factors (e.g., smoking and viral infection) is an important factor in the long-term survival of patients with these compound heterozygous mutants as well as single mutations [3].

**Table 4:** Clinical features of congenital surfactant dysfunction caused by *ABCA3* mutations in the literature

	Literature	Number of cases	Gender	Family history	Mutation type	Mutation site/nucleic acid changes/amino acid changes	Histopathological examination	Histopathological examination methods	Outcome	Survival time	Lung transplantation	Drug treatment
1	Our case	1	M	No	Compound heterozygous	c.1142T > G, c.731G > T	Yes	VAST	Dead	1 mon	No	Surfactant, azithromycin
2	Gupta N P [15]	1	F	No	Homozygous	p.Leu437Pro	Yes	Not available	Dead	3 mon	No	Surfactant, steroid, sildenafil
3	Kröner C [9]	40	30M/10F	Not available	22 homozygous, 18 compound heterozygous	c.578C > G, c.578C > G, c.643C > A, c.863G > A, c.922C > T, c.4164G > C, c.3960G > A, c.4060G > A, c.4195G > A and others	Yes	Not available	11 Survival (5 Homozygous,6 Compound heterozygous), 29 Dead(17 Homozygous,12 Compound heterozygous)	0.1–36 y (median age 0.11 y)	2 (1 Homozygous,1 Compound heterozygous)	Surfactant, steroid, hydroxychloroquine and azithromycin
4	Jouza M [1]	1	F	No	Compound heterozygous	c.440C > T (p.Pro147Leu), c.737C > T (p.Pro246Leu)	Yes	Autopsy	Dead	1 mon	No	Surfactant
5	Wang J [24]	7	3M/4F	Not available	3 homozygous, 4 compound heterozygous	c.3862 + 1G > C, p.R1561X/R1561X, c.3862 + 1G > C, p.P193S/G1412R, p.E1266Q/A1223T, p.P193S/G1412R, p.E1266Q/A1223T, c.1897-1G > C/p.R280H	No	─	7 Dead	1–2 mon (median age 1 mon)	No	Surfactant, steroid, azithromycin
6	Tan JK [10]	1	F	No	Homozygous	c.920C > T	No	─	Survival	1 y	No	Surfactant, steroid
7	Shaaban W [11]	1	M	No	Homozygous	c.875A < T	No	─	Survival	6 mon	No	Surfactant, steroid, hydroxychloroquine and azithromycin
8	Sallmon H [12]	1	F	No	Compound heterozygous	c.2890G > A/c.2125C > T	No	─	Survival	5 mon	No	Surfactant, steroid, hydroxychloroquine, sildenafil, bosentan, L-arginine
9	Akil N [13]	1	M	No	Compound heterozygous	c.875A > T	No	─	Survival	1 y	No	Surfactant, steroid, azithromycin
10	Piersigilli F [26]	2	2M	Yes	Compound heterozygous	c.580A > G	Yes	Autopsy	Dead	3 mon	No	Surfactant, steroid, hydroxychloroquine
11	Oltvai, ZN [27]	1	M	No	Homozygous	c.2883C > T	Yes	Open lung biopsy	Survival	9 mon	Yes	Surfactant, steroid
12	El Boustany P [14]	1	F	No	Compound heterozygous	c.3902del/c.5084_5097del	No	─	Dead	5 y	No	Surfactant, steroid, hydroxychloroquine and azithromycin
13	Cho JG [6]	1	F	Yes	Compound heterozygous	c.127C > T/c.3609_11delCTT	Yes	Open lung biopsy	Survival	39 y	No	Steroid
14	Wei M [28]	1	M	No	Homozygous	c.746C > T	No	─	Dead	2 mon	No	Surfactant, Steroid, hydroxychloroquine, and azithromycin

*ABCA3* ATP-binding cassette subfamily A member 3, *M* male, *F* female, *VAST* video-assisted thoracoscopic surgery

The lung tissue histological results of the infant were similar to pathological alterations previously reported regarding *ABCA3* mutations homozygous or compound heterozygous [9]. In addition, these characteristic pathological changes reduce the diffusional barrier and ventilation–perfusion mismatch, resulting in persistent hypoxemia and refractory carbon dioxide retention.

Whole-exome analysis revealed a compound heterozygote consisting of two *ABCA3* variants. These two variants are located in exons 11 (c.1142T > G) and 8 (c.731G > T) and formed a compound heterozygous (trans) relationship. Both were missense mutations in the parents. Transformation is rare and has not been previously reported. The GnomAD, ExAC, human gene mutation database, and ClinVar databases do not contain these two variations. The c.1142T > G variant is located in exon 11 of ABCA3, resulting in p. Leu381Arg (L381R). The c.731G > T variant is located in exon 8 of ABCA3, resulting in p. Arg244Met (R244M).

In our three-dimention (3D) models, the R244M mutation was located in the ECD1 region, which is an extracellular structural domain. Previous research has shown that missense mutations in the ECD1 region of *ABCA3* lead to the accumulation of ABCA3 in the endoplasmic reticulum, resulting in defective protein synthesis [29]. In contrast, the L381R mutation was located in the TM5 region of the TMD1 domain that, together with other TM proteins, forms a sizable hydrophobic cavity connecting the external environment to the intracellular membrane. Substrates transferred by ABCA3 proteins use these conduits to cross the cell membrane. ECD loops and TMDs are the leading substrate-binding sites that allow the trafficking of lipid molecules [30].

Calculated using the MutPred2 algorithm, the alteration of R244M disrupted the original extracellular membrane orderly interface, where ECD1 was located. The mutation may affect the activity of this ECD1 region in the ABCA3 protein, which contains lipid molecule-binding sites. Moreover, the R244M mutation was speculated to affect the structure of the TM2 transmembrane protein, resulting in alteration of the structural part of the channel occupied by this protein.

The L381R mutation was found in TM5, a partial structural domain of TMD1. TMD1 is in contact with the cytoplasmic end and forms a hydrophobic cavity across the membrane and other structures. Therefore, we hypothesize that the changes in extended strand and β-turn occupancy affected the space of the transmembrane channel structure, and the transformation of the transmembrane channel structure space directly affected the functional ABCA3 protein shift, which may lead to disease.

Based on MutPred2 predictions, we believe that the complex heterozygous mutation located in the structural domains of ECD1 and TMD1 ultimately weakens the transport of substrate lipid molecules, thus reducing the performance of surfactants and eventually leading to CSDD.

This study has some limitations. For example, we did not perform an electron microscopy examination to study LBs and their secretion, and we could not collect samples of the three-generation pedigree and conduct protein functional validation. Furthermore, due to the hash rate limitation, not all residues were involved in the structural modeling of the variants. In addition, the mutation results inferred by the MutPred2 and SOPMA algorithms were not certain to occur but instead represented the likelihood of the effect occurrence. Further research with X-ray diffraction or cryo-electron microscopy is needed.

In conclusion, the novel ABCA3 compound heterozygous mutation resulted in structural changes in the TMD1 and ECD1 regions of ABCA3. They may ultimately weaken the transport of lipid molecules, thus reducing the performance of surfactants and eventually leading to CSDD. The development of genotype–phenotype relationships is often unpredictable in genetic diseases, such as CSDD, caused by compound heterozygous mutations. Therefore, bioinformatics can play an essential role in this class of fields.

## Supplementary Information

Below is the link to the electronic supplementary material.

**Fig. 1 Radiography chest X-ray of the case. a** Postnatal chest X-ray on day 3; **b** postnatal chest X-ray on day 15; **c** postnatal chest X-ray on day 35 (TIF 1224 KB)

**Fig. 2 Computer tomography(CT) scans of the case. a **Patchy shadow in the back of the upper lobes of the right lung; **b** blurred bronchovascular bundles and decreased transparency in both lungs; **c** blurred bronchovascular bundles and decreased transparency in both lungs (TIF 1124 KB)

**Fig. 3 Surface view of the left lung via video-assisted thoracoscopic surgery (VATS).** Uneven inflation and slightly scattered white granules appeared on the surface of the left lung during the operation (TIF 99 KB)

**Fig. 4 Histological examination of the lung tissue. a** Incomplete expanded or collapsed alveolar cavity, increased alveolar basement membrane fibrin (white arrow) (H&E staining; × 100); **b** alveolar compartment was widened (white arrow) (H&E staining; × 200); **c** vascular dysplasia accompanied by inflammatory cell infiltration (white arrow) (H&E staining; × 400); **d** it showed serous exudate (black arrow), tissue reaction in the alveolar, angiodysplasia, and inflammatory cell infiltration in the pulmonary interstitial (white arrow) (H&E staining; × 400); **e** weak CD31 expression in pulmonary vascular endothelial cells (immunostaining; × 200); **f** strong CK expression in alveolar epithelial cells (immunostaining; × 200); **g** weak CD34 expression in pulmonary vascular endothelial cells (immunostaining; × 400); **h** CD68 expression in the alveolar monocytes (immunostaining; × 200)； **i** increased alveolar basement membrane fibrin and the thicken alveolar septa white arrow) (Masson staining; × 200); **j** fibrin deposition around the alveolar cavity white arrow) (PAS staining; × 200). *H&E* hematoxylin–eosin, *CD* cluster of differentiation, *CK* cytokeratin, *PAS* periodic acid-Schiff (TIF 9479 KB)

## Data Availability

The data sets generated during and/or analyzed during the current study are available from the corresponding author on reasonable request.
