# Supplementary figures and images for: Novel insights into congenital surfactant dysfunction disorders by in silico analysis of ABCA3 proteins
Source: World J Pediatr. 2022 Nov 20;19(3):293–301. doi: 10.1007/s12519-022-00645-y (PMC9974682; doi:10.1007/s12519-022-00645-y)

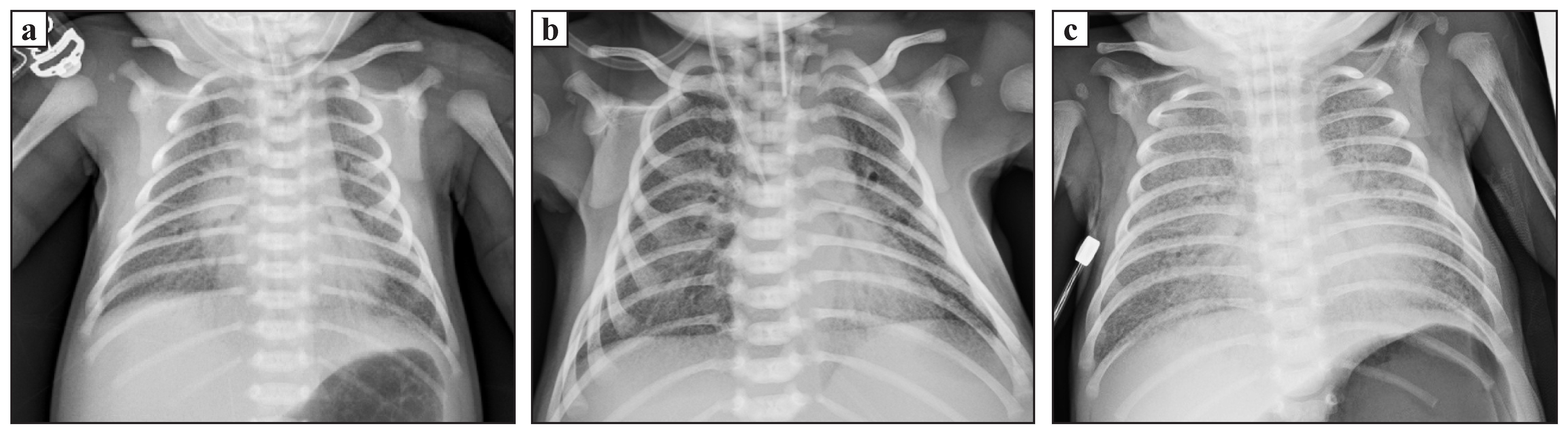

Supplement: Supplementary file 1 — Fig. 1 Radiography chest X-ray of the case. a Postnatal chest X-ray on day 3; b postnatal chest X-ray on day 15; c postnatal chest X-ray on day 35 (TIF 1224 KB) [file 12519_2022_645_MOESM1_ESM.tif]

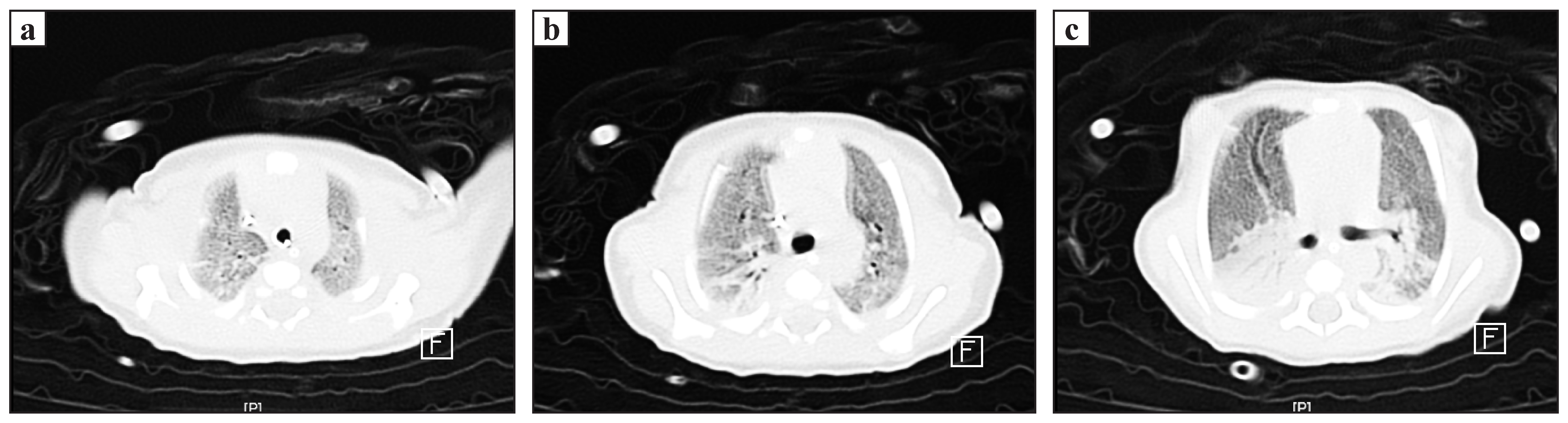

Supplement: Supplementary file 2 — Fig. 2 Computer tomography(CT) scans of the case. a Patchy shadow in the back of the upper lobes of the right lung; b blurred bronchovascular bundles and decreased transparency in both lungs; c blurred bronchovascular bundles and decreased transparency in both lungs (TIF 1124 KB) [file 12519_2022_645_MOESM2_ESM.tif]

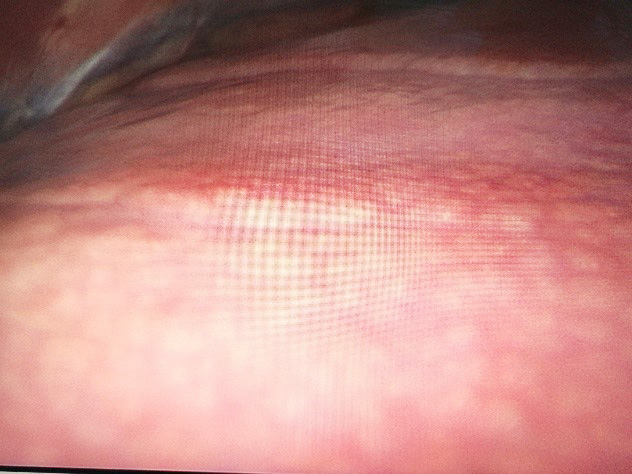

Supplement: Supplementary file 3 — Fig. 3 Surface view of the left lung via video-assisted thoracoscopic surgery (VATS). Uneven inflation and slightly scattered white granules appeared on the surface of the left lung during the operation (TIF 99 KB) [file 12519_2022_645_MOESM3_ESM.tif]

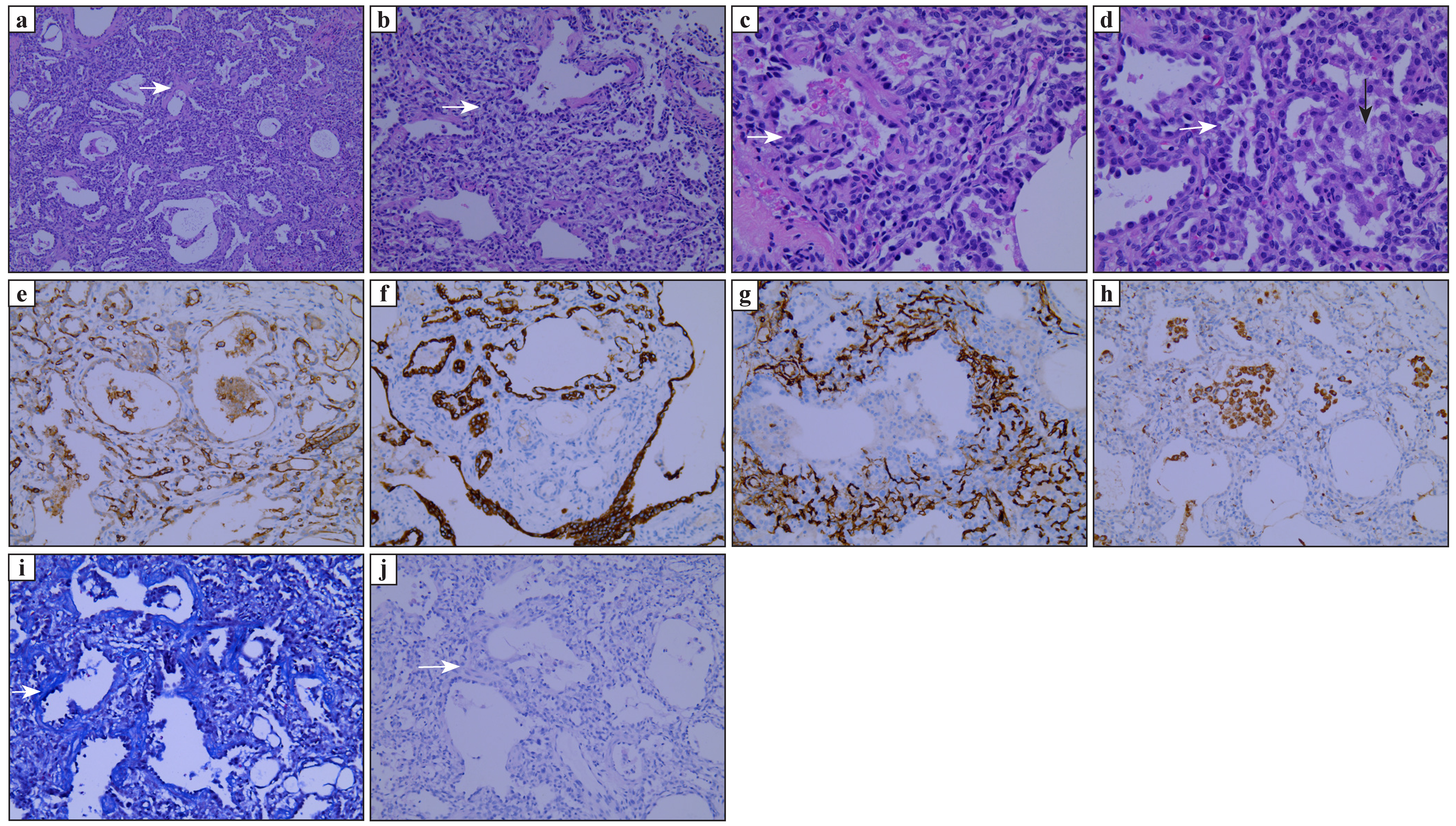

Supplement: Supplementary file 4 — Fig. 4 Histological examination of the lung tissue. a Incomplete expanded or collapsed alveolar cavity, increased alveolar basement membrane fibrin (white arrow) (H&E staining; × 100); b alveolar compartment was widened (white arrow) (H&E staining; × 200); c vascular dysplasia accompanied by inflammatory cell infiltration (white arrow) (H&E staining; × 400); d it showed serous exudate (black arrow), tissue reaction in the alveolar, angiodysplasia, and inflammatory cell infiltration in the pulmonary interstitial (white arrow) (H&E staining; × 400); e weak CD31 expression in pulmonary vascular endothelial cells (immunostaining; × 200); f strong CK expression in alveolar epithelial cells (immunostaining; × 200); g weak CD34 expression in pulmonary vascular endothelial cells (immunostaining; × 400); h CD68 expression in the alveolar monocytes (immunostaining; × 200)； i increased alveolar basement membrane fibrin and the thicken alveolar septa white arrow) (Masson staining; × 200); j fibrin deposition around the alveolar cavity white arrow) (PAS staining; × 200). H&E hematoxylin–eosin, CD cluster of differentiation, CK cytokeratin, PAS periodic acid-Schiff (TIF 9479 KB) [file 12519_2022_645_MOESM4_ESM.tif]
